# Integrative computational approach to farnesyltransferase inhibition toward anti-liver cancer drug candidate from *Syzygium cumini* essential oils

**DOI:** 10.22099/mbrc.2025.54110.2201

**Published:** 2026

**Authors:** Wira Eka Putra, Arief Hidayatullah, Diana Widiastuti, Hary Isnanto, Muhammad Fikri Heikal, Sustiprijatno

**Affiliations:** 1 Biotechnology Study Program, Department of Applied Sciences, Faculty of Mathematics and Natural Sciences, Universitas Negeri Malang, East Java, 65145, Indonesia; 2 Democratic Governance and Poverty Reduction Unit, United Nations Development Programme, Eijkman-RSCM Building, Jakarta, 10430, Indonesia; 3 Department of Chemistry, Faculty of Mathematics and Natural Science, Universitas Pakuan, West Java, 16129, Indonesia; 4 Department of Biochemical Technology, School of Bioresources and Technology, King Mongkut's University of Technology Thonburi, Bangkok, 10140, Thailand; 5 Tropical Medicine Graduate Program, Faculty of Medicine, Khon Kaen University, Khon Kaen, 40002, Thailand; 6 Research Center for Applied Botany, National Research and Innovation Agency, West Java, 16911, Indonesia

**Keywords:** In silico, Metastasis, MMP9, Natural products, S. cumini

## Abstract

Farnesyltransferase plays a critical role in the post-translational modification of mammalian proteins, including the Ras oncogene, which is strongly associated with cancer development. Essential oils from *Syzygium cumini* have demonstrated promising therapeutic effects, particularly in cancer treatment, potentially through the inhibition of farnesyltransferase activity. This study employed integrative computational approaches to investigate the anticancer potential of essential oils derived from *S. cumini*. Various compounds were screened for toxicity, biological activities, membrane permeability, gene expression profiles, and survival correlations were conducted to investigate cancer-associated properties. Molecular docking and molecular dynamics (MD) simulations were performed to evaluate the binding interactions and stability of ligand–protein complexes involving farnesyltransferase. α-Humulene epoxide II exhibited antineoplastic activity, functioned as an apoptosis agonist, and inhibited cancer-related targets such as HIF1A and MMP9. Bornyl acetate showed potential as a JAK2 inhibitor. Both compounds demonstrated favorable membrane permeability, indicating high bioavailability and effective cellular uptake. Analysis of the farnesyltransferase (FNTB) gene revealed significantly higher expression in cancerous tissues and a positive correlation with pro-tumor immune cell infiltration. Molecular docking identified Tipifarnib as the strongest binder, serving as a positive control, while α-humulene epoxide II and bornyl acetate showed moderate to weaker binding affinities. However, MD simulations confirmed that both essential oil compounds exhibit binding stability comparable to that of Tipifarnib. Finally, α-humulene epoxide II and bornyl acetate from *S. cumini* exhibit favorable drug-like properties, high predicted safety margins, and a lack of organ-specific toxicity, underscoring their suitability for further drug development.

## INTRODUCTION

Farnesyltransferase belongs to the protein prenylation family and is essential for the post-translational modification of mammalian proteins, facilitating the attachment of isoprenoid lipids under normal physiological conditions [1]. Emerging evidence indicates that farnesyl-transferase is involved in various human diseases, including glaucoma, neurological disorders, infectious diseases, and cancer. Notably, farnesyltransferase and geranylgeranyltransferase play a critical role in the post-translational modification of Ras proteins, which are well-established oncogenes [2]. Inhibiting farnesyltransferase activity can prevent Ras oncoprotein activation, disrupting downstream signaling pathways associated with tumor initiation and progression [3]. Therefore, the development of farnesyltransferase inhibitors represents a promising strategy for cancer treatment.

Several studies have demonstrated the effectiveness of farnesyltransferase inhibition, leading to positive clinical outcomes in cancer patients. For instance, the combination of targeted therapy with farnesyltransferase inhibitors has been shown to prevent tumor relapse and enhance long-term treatment responses in oncogene-addicted non-small cell lung cancer patients [4]. Additionally, the combination of farnesyltransferase inhibitors with KRAS G12C inhibitors has demonstrated synergistic antitumor effects in cellular models of lung, colorectal, and pancreatic adenocarcinomas [5]. Their clinical impact is promising given the therapeutic potential of farnesyltransferase inhibitors as blocking agents in cancer treatment. However, current farnesyltransferase inhibitors still face challenges, particularly concerning toxicity, safety, and efficacy. Therefore, identifying natural product-derived inhibitors represents a compelling avenue for further research, as they may offer a safer, low-toxicity, and effective alternative for cancer therapy [6]. 

Plant-derived natural products have demonstrated significant potential as a valuable source for drug discovery and development [7]. *S. cumini*, commonly known as black plum, is a medicinal plant from the Myrtaceae family, recognized for its therapeutic effects against various ailments, including diabetes, hypertension, obesity, hyperlipidemia, inflammation, anemia, bacterial infections, oxidative stress, allergies, and liver disorders [8, 9]. Moreover, *S. cumini* has shown promising potential as an anti-cancer agent. For instance, its ethanolic extract has been reported to suppress the growth of HT-29 colorectal cancer cells [10]. The methanolic extract has also demonstrated anti-proliferative and pro-apoptotic effects in Ehrlich Ascites Carcinoma cells [11].

*S. cumini* contained a high number of bioactive compounds, which contribute to its therapeutic properties. It has been reported to contain various essential oil components, including α-pinene, β-pinene, D-limonene, α-terpineol, bornyl acetate, trans-β-caryophyllene, α-caryophyllene, γ-muurolene, (+)-spathulenol, caryophyllene oxide, and α-humulene epoxide II [12]. In this study, we hypothesize that specific bioactive compounds from *S. cumini* may exhibit anti-cancer activity by inhibiting farnesyltransferase. Therefore, this study aims to explore the potential of *S. cumini* essential oils as farnesyltransferase inhibitors through an integrative computational approach, with the goal of identifying novel anti-liver cancer drug candidates. 

## MATERIALS AND METHODS

### Virtual screening and toxicity prediction:

Essential oil compounds from *S. cumini*, such as α-pinene, β-pinene, D-limonene, α-terpineol, bornyl acetate, trans-β-caryophyllene, α-caryophyllene, γ-muurolene, (+)-spathulenol, caryophyllene oxide, and α-humulene epoxide II, were virtually screened in order to find and filter possible drug candidates. The drug-likeness properties of these compounds were assessed based on multiple criteria, including Lipinski’s, Ghose’s, Veber’s, and Egan’s rules. The compounds that did not violate any of these criteria were considered to possess drug-like properties. Virtual screening was conducted using SwissADME (http://www.swissadme.ch) [13]. Subsequently, the selected compounds were evaluated for toxicity based on several parameters, including toxicity classification, predicted LD50, and the probability of inducing organ toxicity. Toxicity prediction was performed using ProTox 3.0 (https://tox.charite.de/protox3) [14].

### Biological activity and membrane permeability prediction:

The selected compounds with drug-like properties and minimal predicted toxicity were further evaluated for their biological activity, particularly in relation to cancer-associated properties such as anticarcinogenic, antineoplastic, apoptosis agonist, and inhibition of BRAF, HIF1A, JAK2, MMP9, Myc, NOS2, Pin1, and TNF. Biological activity prediction was conducted using the Way2Drug web server (https://www.way2drug.com/passonline) [15]. Additionally, membrane permeability prediction was performed for the selected compounds, as this is a crucial factor in drug discovery to determine the potential of a compound to penetrate the plasma membrane effectively. Membrane permeability prediction was carried out using the PerMM server and database (https://permm.phar.umich.edu) under experimental conditions of 310 K and pH 7.4, which align with physiological conditions [16]. 

### Enrichment analysis:

Gene expression analysis, overall survival, and disease-free survival analysis of the FNTB were evaluated using GEPIA (http://gepia.cancer-pku.cn) [17]. Finally, correlation analysis was performed between gene expression of FNTB and immune/stromal cell infiltration, including monocytes, macrophages, neutrophils, regulatory T cells, myeloid-derived suppressor cells (MDSCs), cancer-associated fibroblasts (CAFs), cytotoxic T cells, and natural killer (NK) cells. This analysis was conducted using TIMER 2.0 (http://timer.cistrome.org) [18].

### Molecular docking and molecular dynamics simulation:

 The three-dimensional (3D) structure was obtained from the RCSB PDB database (https://www.rcsb.org/) following the identification of the particular target protein [19]. The human farnesyltransferase protein, PDB ID 1SA4, was used for this investigation. SAVESv6.1 (https://saves.mbi.ucla.edu) and ProSA-web (https://prosa.services.came.sbg.ac.at/prosa.php) were used in a validation procedure to guarantee the structural quality of the retrieved protein [20]. 

Subsequently, molecular docking was conducted between the selected ligands and the target protein. The chemical structures of the selected compounds and the control drug were obtained from the PubChem database (https://pubchem.ncbi.nlm.nih.gov/) [21]. Tipifarnib (CID: 159324) was used as the control drug, as it is a known farnesyltransferase inhibitor [22]. Molecular docking was performed using PyRx 0.8 (https://pyrx.sourceforge.io/) to predict the binding affinity and interactions [23].

Following docking, MD simulations were carried out to assess the stability of the ligand–protein interactions. Simulations were conducted under physiological conditions, including a pH of 7.4, 0.9% NaCl concentration, a temperature of 310 K, a water density of 0.997 g/cm³, and a pressure of 1 atm. Various structural and energetic parameters were analyzed, including the root mean square deviation (RMSD) of the protein backbone, ligand movement, ligand conformation, root mean square fluctuation (RMSF), radius of gyration (Rg), solvent-accessible surface area (SASA), hydrogen bonding (H-bond) interactions, binding energy, and the dynamic cross-correlation matrix (DCCM). MD simulations were performed using YASARA software (https://www.yasara.org) [24], while molecular docking and simulation results were visualized using BIOVIA Discovery Studio Visualizer (https://www.3ds.com).

## RESULTS

**Figure 1:**
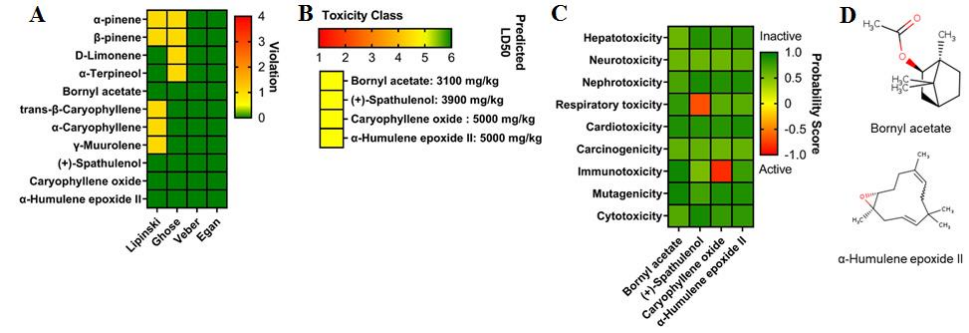
*S. cumini* essential oils were analyzed through virtual screening and toxicity evaluation. (A) Drug-likeness assessment. (B) Toxicity classification and LD50 estimation. (C) Prediction of organ-specific toxicity. (D) Visualization of selected bioactive compounds with promising drug-likeness properties and minimal toxicity risk.

The bioactive compounds from *S. cumini* essential oils were evaluated based on key drug-likeness rules, including those proposed by Lipinski, Ghose, Veber, and Egan. Four of the eleven compounds assessed demonstrated promising drug-like properties: bornyl acetate, (+)-spathulenol, caryophyllene oxide, and α-humulene epoxide II (Fig. 1A). Compounds that exhibited drug-like properties were considered to have favorable pharmacokinetic characteristics, while those violating any of these rules were excluded from further analysis.

Subsequently, these four selected compounds underwent toxicity prediction assessments, including toxicity classification, predicted LD50, and organ-specific toxicity evaluation. The results indicated that all compounds fell under class five in the toxicity classification system, suggesting low toxicity. Regarding LD50 predictions, the compounds exhibited varying values: bornyl acetate (3100 mg/kg), (+)-spathulenol (3900 mg/kg), caryophyllene oxide, and α-humulene epoxide II (5000 mg/kg) (Fig. 1B). Further organ-specific toxicity predictions revealed that (+)-spathulenol had the potential to induce respiratory toxicity, while caryophyllene oxide exhibited a tendency to cause immunotoxicity. 

**Figure 2:**
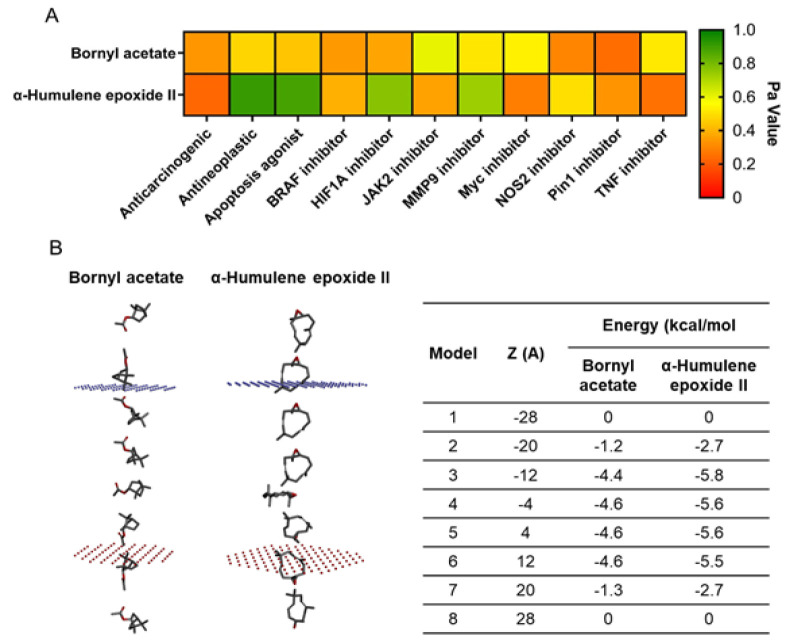
Assessment of the biological activity and membrane permeability of the selected compounds. (A) Predicting biological activity. (B) Evaluating membrane permeability.

In contrast, bornyl acetate and α-humulene epoxide II showed no organ-specific toxicity across the evaluated parameters (Fig. 1C). Based on virtual screening and toxicity evaluation results, bornyl acetate and α-humulene epoxide II were identified as the most promising drug candidates (Fig. 1D) and were selected for further evaluation. The biological activity prediction of the two selected compounds, bornyl acetate and α-humulene epoxide II, was evaluated, particularly focusing on their potential anticancer properties. The results indicated that α-humulene epoxide II exhibited high potency as an antineoplastic, apoptosis agonist, and an inhibitor of HIF1A and MMP9. Meanwhile, bornyl acetate showed strong potential as a JAK2 inhibitor (Fig. 2A). 

Another crucial parameter in drug discovery and development is assessing the ability of compounds to penetrate the plasma membrane. The evaluation results demonstrated that both compounds possess membrane permeability, with their orientation perpendicular to the membrane, indicating potential penetration depth (Fig. 2B). Notably, both bornyl acetate and α-humulene epoxide II exhibited their lowest energy levels between -12 Å and 12 Å, suggesting favourable interactions within the hydrophobic core of the membrane.

**Figure 3:**
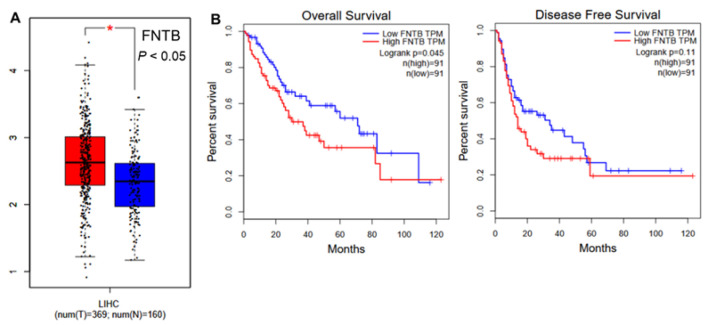
Enrichment analysis using publicly available data. **(A)** Comparative evaluation of FNTB gene expression in liver cancer and normal tissues. **(B)** Analysis of overall survival and disease-free survival in relation to FNTB gene expression.

Gene expression analysis of FNTB in liver hepatocellular carcinoma (HCC) samples demonstrated significantly higher expression in tumor tissues compared to normal tissues (*p*<0.05), indicating its potential relevance in cancer progression (Fig. 3A). Interestingly, the overall survival (OS) curve shows that patients with high FNTB expression have a poorer survival outcome compared to those with low FNTB expression. This difference is statistically significant, as indicated by the log-rank p-value of 0.045, suggesting that FNTB may be associated with worse prognosis in terms of overall survival (Fig. 3B). The disease-free survival (DFS) curve also shows a trend toward poorer outcomes in patients with high FNTB expression, although the difference is not statistically significant (log-rank p=0.11) (Fig. 3B). 

The non-significant trend in DFS suggests that FNTB may be more predictive of mortality than recurrence, or that additional factors modulate its impact on disease-free outcomes. Interestingly, FNTB expression was positively correlated with pro-tumor immune cells, including macrophages, neutrophils, regulatory T cells, myeloid-derived suppressor cells (MDSCs), and cancer-associated fibroblasts (CAFs). These imply that elevated FNTB levels may correlate with enhanced infiltration of these immunosuppressive cells. Conversely, FNTB showed no significant correlation with monocytes, cytotoxic T cells or natural killer (NK) cells, which are known for their anti-tumor activity (Fig. S1). Therefore, these findings indicates that FNTB may not exert a significant direct influence on adaptive anti-tumor immunity.

The Ramachandran plot analysis indicated that 92.1% of residues fell within the most favored regions, reflecting good stereochemical quality. Additionally, 7.7% of residues were in allowed regions, while 0.2% were in generously allowed regions, suggesting minor deviations. Importantly, no residues (0.0%) were located in disallowed regions, confirming a well-refined model (Fig. S2A). Further validation using ProSA showed a Z-score of -8.76, indicating high structural quality compared to experimentally determined structures. The knowledge-based energy plot also demonstrated predominantly negative values across sequence positions, suggesting good structural stability (Fig. S2B). The ERRAT plot revealed an overall quality factor of 96.888, further supporting the model's reliability (Fig. S2C). Lastly, the Verify 3D plot yielded a quality assessment score of 83.86%, indicating high structural consistency and reliability (Fig. S2D).

**Figure 4:**
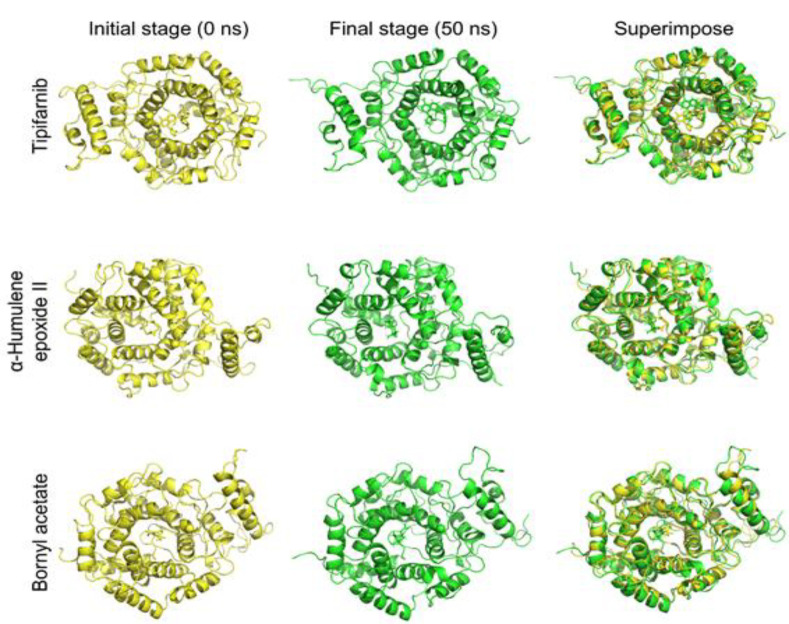
Analysis of the structural flexibility and conformational variations of the protein-ligand complex throughout molecular dynamics simulation.

Molecular docking analysis evaluated the binding interactions of three ligands with the farnesyltransferase. The docking results demonstrated that all three ligands occupied the same binding site, with a zoomed-in view highlighting their overlapping binding orientations (Fig. S3A). The docked ligands included Tipifarnib (black), α-humulene epoxide II (red), and bornyl acetate (blue), indicating comparable binding site preferences. A bar graph representation of binding affinity predictions (kcal/mol) showed that Tipifarnib exhibited the strongest binding (-9.2 kcal/mol), serving as a positive control (Fig. S3B). α-Humulene epoxide II demonstrated moderate binding affinity (-7.3 kcal/mol), suggesting its potential as a farnesyltransferase inhibitor. In comparison, bornyl acetate showed the weakest binding (-5.7 kcal/mol), indicating lower affinity for the farnesyltransferase. The MD snapshots revealed some positional changes in the protein and ligands, indicating molecular movement during interactions (Fig. 4). Despite this, all of the target compounds remained in their respective binding sites throughout the simulation.

**Figure 5:**
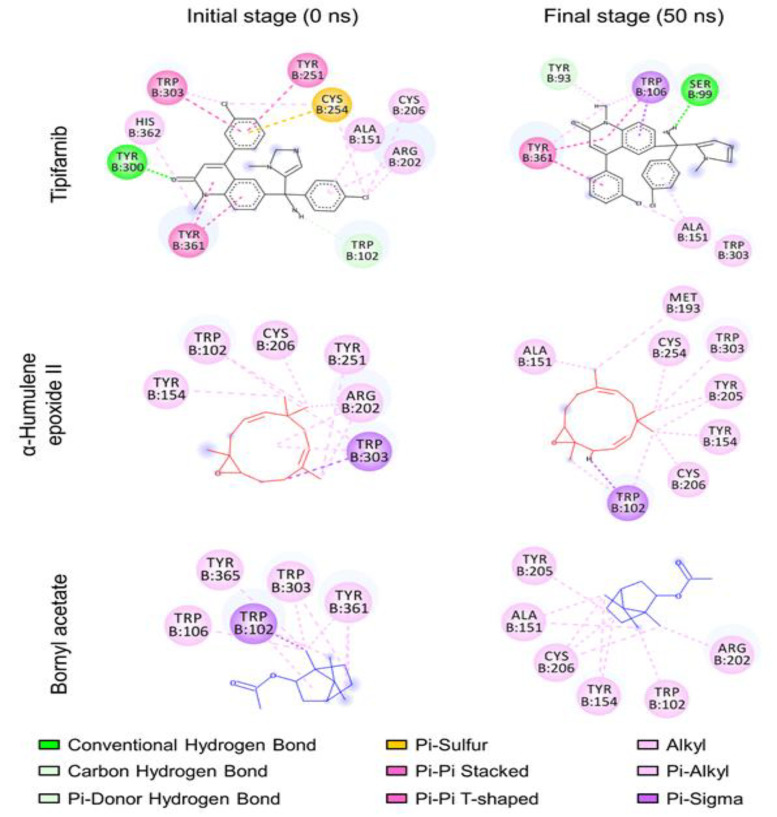
Evaluation of ligand dynamics and conformational shifts during molecular dynamics simulation, focusing on changes in chemical interactions.

Furthermore, an analysis of chemical interactions demonstrated shifts in binding patterns over the course of the simulation (Fig. 5). At the initial stage, the farnesyltransferase–Tipifarnib complex formed hydrogen bonds with Tyr300 and Trp102. By the final stage, the ligand maintained several key interactions; however, some hydrogen bonds shifted, forming new interactions with Ser99 and Tyr93, replacing the original bonds with Tyr300 and Trp102. These findings suggest a stable binding pattern with minor interaction rearrangements. In contrast, α-humulene epoxide II and bornyl acetate primarily engaged in hydrophobic interactions. Notably, their interaction patterns shifted significantly by the end of the simulation, indicating weaker binding stability compared to the control drug, Tipifarnib.

**Figure 6:**
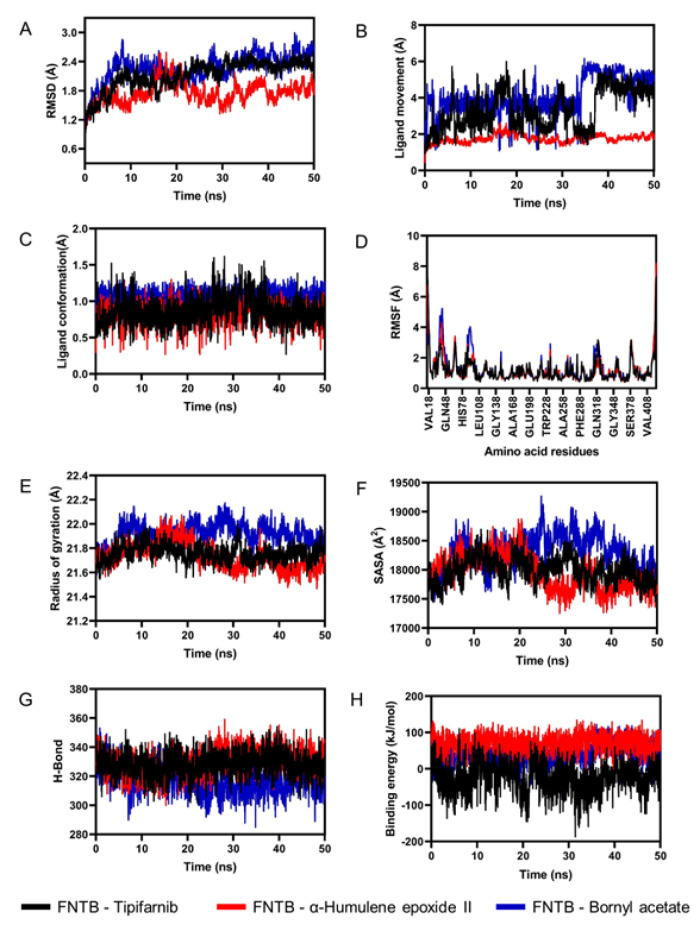
Molecular dynamics simulation of ligands with the target protein FNTB. (A) Backbone RMSD plot. (B) Ligand movement RMSD plot. (C) Ligand conformation RMSD plot. (D) RMSF plot. (E) Radius of gyration plot. (F) SASA plot. (G) Hydrogen bond interaction plot. (H) Binding energy plot.

We analyzed key parameters during the 50 ns MD simulation to assess protein–ligand complex stability. RMSD of the protein backbone showed Tipifarnib fluctuated moderately (approximately 2.4 Å), bornyl acetate had the highest RMSD indicating instability, and α-humulene epoxide II had the lowest, suggesting the most stable binding (Fig. 6A). Ligand RMSD revealed α-humulene epoxide II remained stable (approximately 2.0 Å), while Tipifarnib and bornyl acetate showed greater fluctuations (Fig. 6B). Tipifarnib stabilized in the last 10 ns, and bornyl acetate in the last 15 ns. Ligand conformation and RMSF plots indicated similar trends and overall conformational stability (Fig. 6C and 6D). 

**Figure 7:**
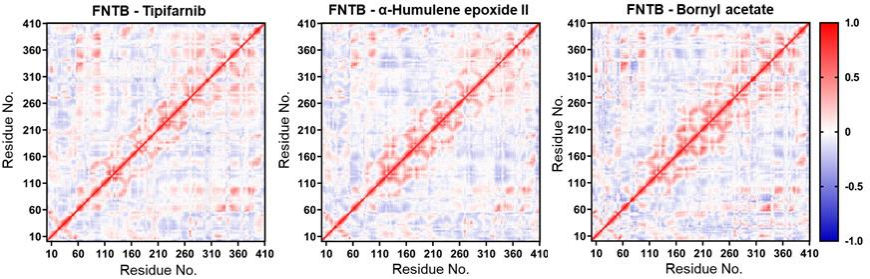
Dynamic cross-correlation matrix analysis of the FNTB-ligand complex

Radius of gyration analysis showed that Tipifarnib and α-humulene epoxide II maintained compact structures, with slight contraction in the latter, while bornyl acetate had higher, fluctuating Rg values, indicating reduced stability (Fig. 6E). SASA analysis revealed higher solvent exposure for bornyl acetate and lower exposure for the other two, suggesting stronger binding by Tipifarnib and α-humulene epoxide II (Fig. 6F). Hydrogen bond analysis supported this, with bornyl acetate forming fewer bonds (Fig. 6G). Binding energy results showed Tipifarnib had the strongest affinity, followed by α-humulene epoxide II and bornyl acetate (Fig. 6H). DCCM analysis indicated distinct dynamic effects for bornyl acetate (Fig. 7).

## DISCUSSION

Research on terpenoids is gaining significant attention in cancer studies. This investigation explores the potential anticancer properties of two terpenoids, namely α-humulene epoxide II and bornyl acetate. While limited studies specifically address these compounds, existing evidence highlights the cytotoxic effects of α-humulene, a sesquiterpene hydrocarbon. It induces mitochondrial dysfunction, disrupts membrane potential, promotes oxidative stress, and triggers mitochondrial-dependent apoptosis by activating pro-apoptotic proteins like Bax and inhibiting anti-apoptotic Bcl-2 [25, 26]. α-Humulene also exhibits antiproliferative activity against various cancer cell lines, including colorectal, hepatic, ovarian, and colon adenocarcinoma cells [25]. In contrast, bornyl acetate, a bicyclic monoterpene, is recognized for its strong anti-inflammatory and anticancer effects. It inhibits pro-inflammatory cytokines such as TNF-α and IL-1β, reduces myeloperoxidase activity, and suppresses the NF-κB and MAPK signaling pathways by blocking phosphorylation of IKB, IKK, ERK, JNK, and p38 [27, 28]. Additionally, it induces G1 cell cycle arrest in colorectal cancer via inhibition of NF-κB and COX-2 [29]. 

The FNTB gene encodes farnesyltransferase subunit beta (FTase subunit β), predominantly featuring the α-helical structure that forms a central barrel hosting a single zinc ion. This ion coordinates the thiol group of the Ras oncoprotein's CaaX motif during farnesylation [30, 31]. FNTB's peptide-binding domain preferentially recognises Ras GTPases, particularly HRAS, which relies on farnesylation [32]. The FTase’s α-subunit stabilises the structure, while the β-subunit facilitates the binding of farnesyl diphosphate and transfers the farnesyl group. The FNTB α-helical structure forms a hydrophobic cavity for farnesyl diphosphate (FPP) but not geranylgeranyl pyrophosphate (GGPP) [33].

FNTB’s hydrophobic cavity contains conserved residues like Trp102, Trp106, and Trp303, providing a π-electron-rich environment stabilising the farnesyl chain of FPP through van der Waals interactions. Phe253 and Phe302 enhance hydrophobicity with their nonpolar side chains, securing the isoprenoid substrate. Tyrosine residues (Tyr105, Tyr154, Tyr205, Tyr361, Tyr365) along the cavity walls may contribute to subtle electrostatic interactions while preserving the hydrophobic nature needed for FPP binding. Key functional residues in FNTB's active site stabilise and catalyse the substrate. Arg202 interacts with the diphosphate group of FPP, stabilizing its orientation for efficient catalysis. A zinc ion, coordinated by Cys299 and other conserved residues like His362 and Asp359, polarizes FPP's thiolate group, enhancing nucleophilic attack on the farnesyl group, crucial for post-translational modification of target proteins [34]. 

Molecular docking studies reveal that α-humulene epoxide II and bornyl acetate exhibit lower binding affinity than Tipifarnib but still occupy the same hydrophobic binding cavity. Due to their simpler molecular structures, these terpenoids interact with fewer residues compared to Tipifarnib. Their compact size enables them to fit within the cavity’s selective dimensions. α-Humulene epoxide II primarily engages in stable hydrophobic and π-alkyl interactions with Trp102, Trp303, and Tyr154 via its cyclic sesquiterpene structure. This suggests a potential inhibitory mechanism involving partial displacement of FPP’s farnesyl chain while sparing the diphosphate-binding region [35]. In contrast, bornyl acetate, defined by its compact bicyclic monoterpene scaffold, undergoes significant interaction shifts during simulation. Initially binding to Trp106, Trp303, and Tyr361, it later associates with Arg202 and Cys206, indicating lower interaction stability [36]. This flexibility may reduce its inhibitory potency, as it lacks sustained interaction with key catalytic residues. This is consistent with studies showing that bornyl acetate modulates signaling pathways such as PI3K/AKT through indirect suppression mechanisms rather than stable catalytic site binding [37]. The differing binding behaviors reflect their inhibitory strengths: Tipifarnib directly competes with FPP via strong, stable catalytic interactions, while the terpenoids likely function as weaker allosteric modulators, inducing subtle conformational changes that affect substrate binding or catalysis [38].

The dynamic simulations suggest that α-humulene epoxide II maintained exceptional stability throughout the simulation, which aligns with studies demonstrating that ligands inducing minimal backbone fluctuations often form more stable interactions with target proteins [39]. Tipifarnib and bornyl acetate exhibited some general fluctuations, indicating transient binding conformations despite maintained active-site occupancy. This discrepancy may arise from differential interaction modes, as evidenced by hydrogen bond analysis [40]. The Rg, SASA, and superimposed structural analyses further suggest that, although all could maintain their respective complexes intact, Tipifarnib and α-humulene epoxide II could maintain better structural integrity, implicating binding stability. The DCCM analysis revealed that bornyl acetate induced distinct correlated motions in FTase compared to Tipifarnib and α-humulene epoxide II, suggesting ligand-specific modulation of protein dynamics. Such dynamic perturbations may influence FTase's ability to bind FPP or catalyze farnesyl transfer, as conformational flexibility is essential for enzymatic activity [41]. 

This study highlights the anticancer potential of α-humulene epoxide II and bornyl acetate from *S. cumini *essential oil as natural FNTB-targeting agents. Both terpenoids exhibit favorable drug-like and safety profiles. Molecular docking and MD simuation suggest α-humulene epoxide II binds stably via hydrophobic and π-alkyl interactions, while bornyl acetate interacts more flexibly. Though less potent than Tipifarnib, the compounds may act as allosteric modulators, subtly affecting FNTB activity. Structural and dynamic analyses support their distinct influence on protein behavior. These findings position the terpenoids as promising scaffolds for developing novel farnesyltransferase inhibitors, warranting further in vitro and in vivo validation.

### Acknowledgements:

The authors thank Universitas Negeri Malang for supporting this study. This research received no specific grant from any funding agency in the public, commercial, or not-for-profit sectors.

### Conflict of Interest:

The authors declare that there are no conflicts of interest related to this article. 

### Authors’ Contribution:

Conceptualization: WEP, AH, DW; Data curation: WEP, AH, HI, MFH; Formal analysis: WEP, AH, DW, HI, MFH, S; Funding acquisition: WEP; Investigation: WEP, AH, DW, HI, MFH, S; Methodology: WEP, AH, DW, HI, MFH, S; Project administration: WEP, MFH, S; Resources: WEP, MFH, S; Software: WEP, AH, HI, MFH; Supervision: WEP, DW, S; Validation: WEP, AH, DW, HI, MFH, S; Visualization: WEP, AH, DW, MFH; Writing - original draft: WEP, AH, DW, HI; Writing - review and editing: MFH, S. All authors read and approved the final manuscript.
