# Supplementary material for: Integrative computational approach to farnesyltransferase inhibition toward anti-liver cancer drug candidate from Syzygium cumini essential oils
Source: Mol Biol Res Commun. 2026;15(1):49–61. doi: 10.22099/mbrc.2025.54110.2201 (PMC12673631; doi:10.22099/mbrc.2025.54110.2201)
Supplement: Supplementary file 1 — Figures S1-S3 [file mbrc-15-49-s001.pdf]

Molecular Biology Research Communications 2026;15(1):49-61 DOI:10.22099/mbrc.2025.54110.2201 MBRC

Original Article Open Access

Integrative computational approach to farnesyltransferase inhibition toward anti-liver cancer drug candidate from *Syzygium cumini* essential oils

Wira Eka Putra[1,*], Arief Hidayatullah[2], Diana Widiastuti[3], Hary Isnanto[4], Muhammad Fikri Heikal[5], Sustiprijatno[6]

1) Biotechnology Study Program, Department of Applied Sciences, Faculty of Mathematics and Natural Sciences, Universitas Negeri Malang, East Java, 65145, Indonesia
2) Democratic Governance and Poverty Reduction Unit, United Nations Development Programme, Eijkman-RSCM Building, Jakarta, 10430, Indonesia
3) Department of Chemistry, Faculty of Mathematics and Natural Science, Universitas Pakuan, West Java, 16129, Indonesia
4) Department of Biochemical Technology, School of Bioresources and Technology, King Mongkut's University of Technology Thonburi, Bangkok, 10140, Thailand
5) Tropical Medicine Graduate Program, Faculty of Medicine, Khon Kaen University, Khon Kaen, 40002, Thailand
6) Research Center for Applied Botany, National Research and Innovation Agency, West Java, 16911, Indonesia

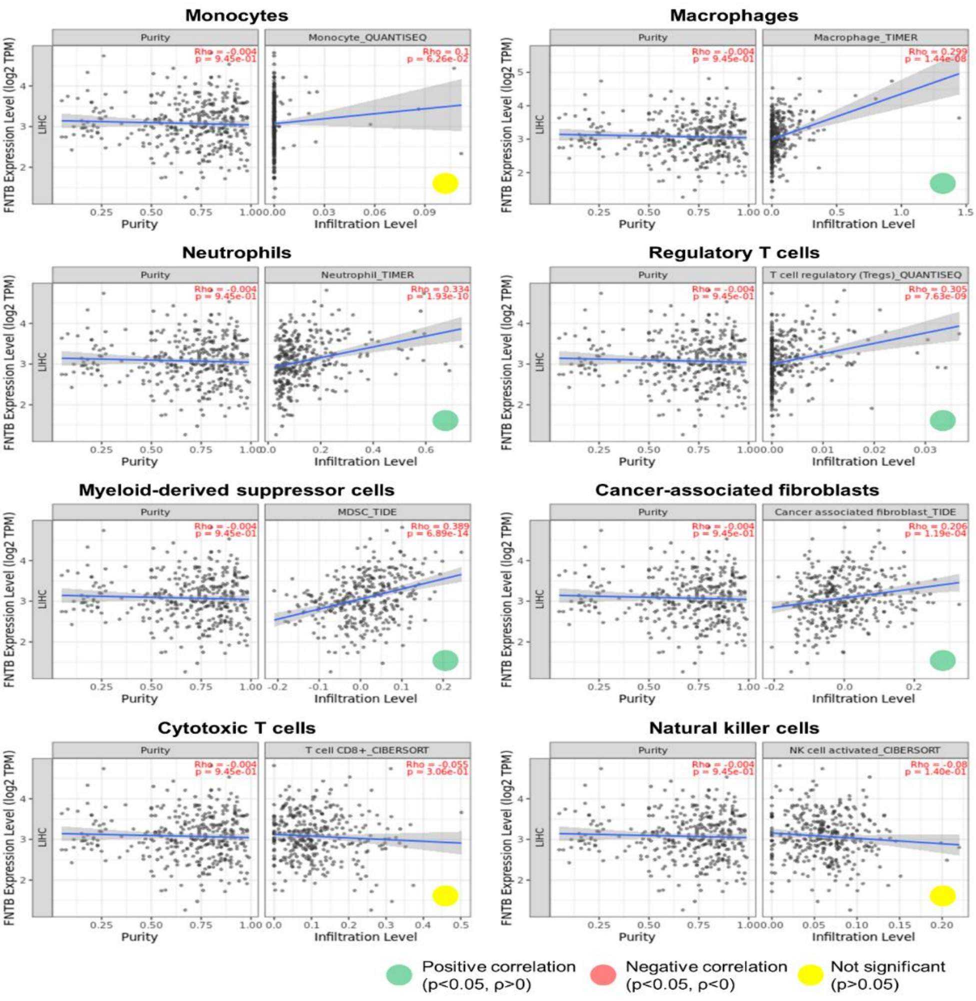

Figure S1: Analysis of the correlation between FNTB gene expression and tumor microenvironment component infiltration in liver cancer patients using publicly available datasets.

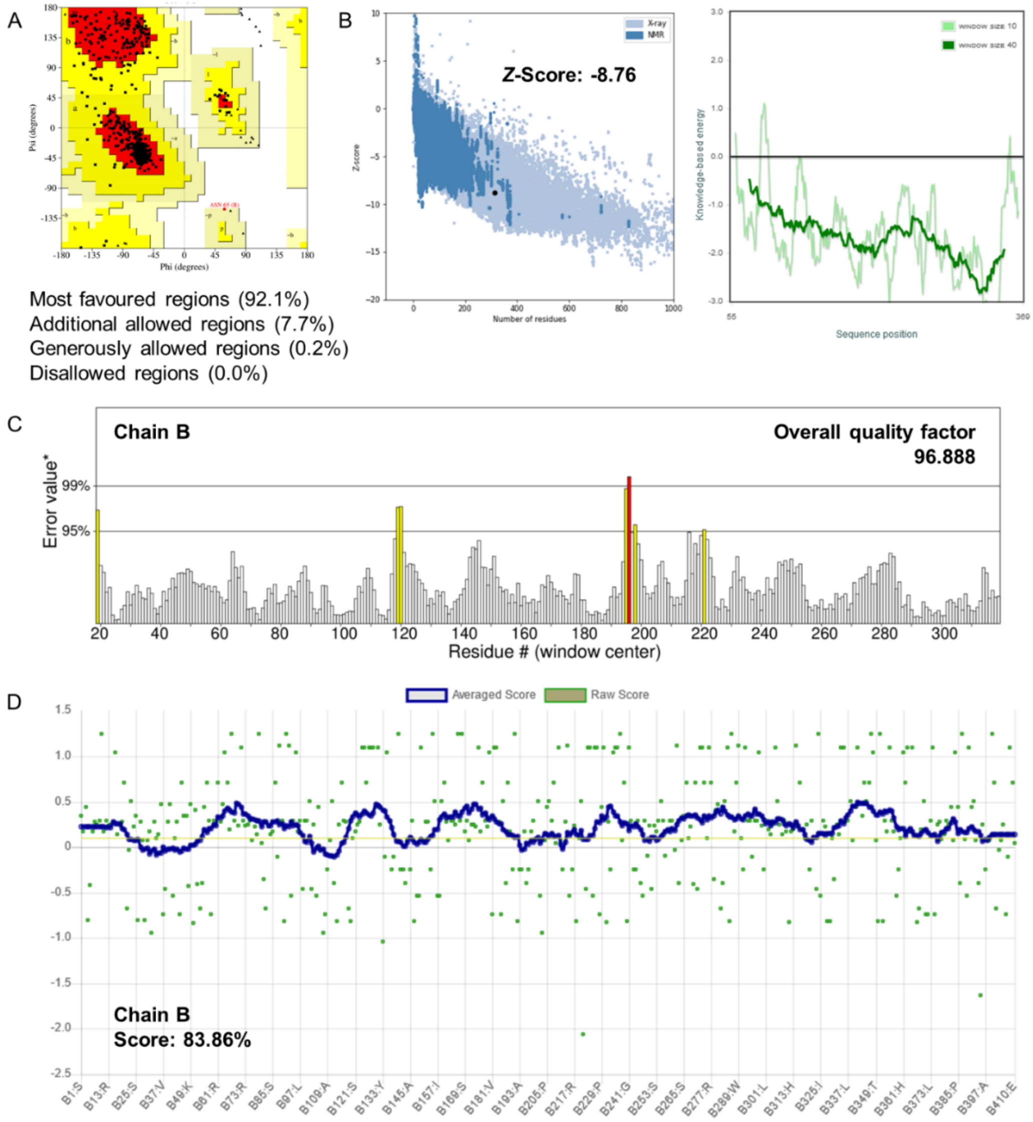

Figure S2: Validation of the FNTB protein structure using various assessment web-tools. (A) Ramachandran plot analysis via SAVES. (B) Protein structure evaluation through ProSA-web. (C) ERRAT plot assessment using SAVES. (D) VERIFY 3D plot analysis via SAVES.

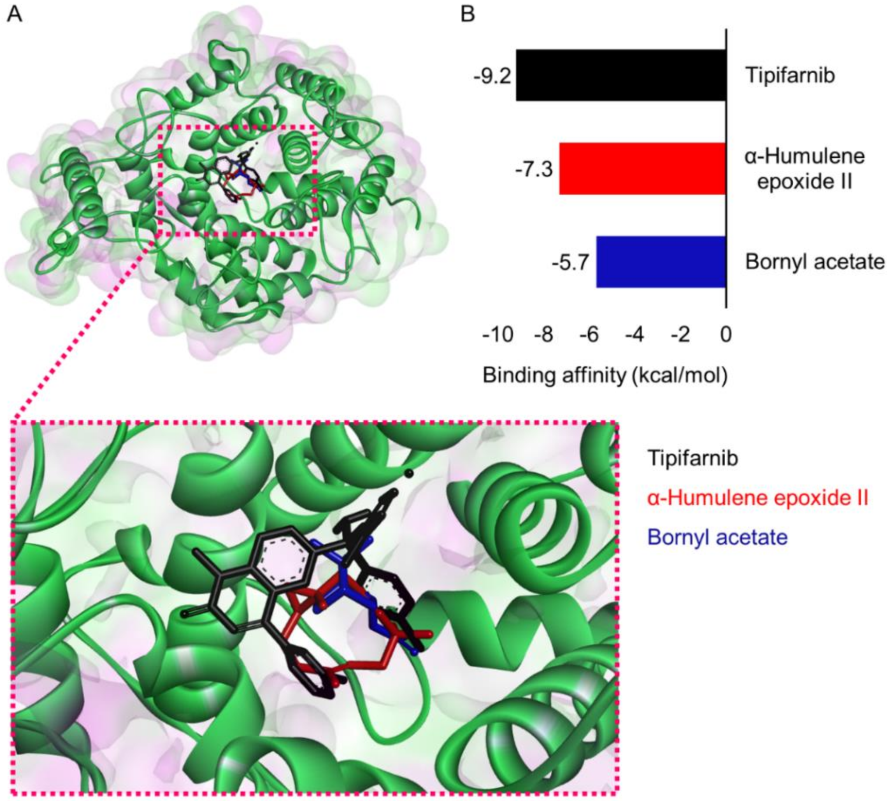

Figure S3: Docking analysis of ligand binding with the target protein. (A) Evaluation of binding affinity scores. (B) 3D visualization of the FNTB-ligand complex.
